# Small-Town America’s Despair: Infected Substance Users Needing Outpatient Parenteral Therapy and Risk Stratification

**DOI:** 10.7759/cureus.1579

**Published:** 2017-08-18

**Authors:** Ulas M Camsari, Claudia R. Libertin

**Affiliations:** 1 Department of Psychiatry & Psychology, Mayo Clinic, Rochester, MN, USA; 2 Internal Medicine, Mayo Clinic, Jacksonville, FL

**Keywords:** : ivdu and opat, risk stratification of ivdu and opat, rural medicine, rural opat care, small town plights

## Abstract

Background

An active intravenous substance use disorder is often the primary cause of infectious diseases in this population of users and creates a barrier to successful parenteral antimicrobial management. The dilemma is compounded by dramatically limited resources in small US towns.

Methods

This retrospective review from January 2014 through July 2016 aimed to develop a risk stratification approach to aid rural healthcare providers in determining who among patients with addictive disorders could safely be discharged for outpatient antimicrobial therapy with a peripherally inserted central catheter (PICC).

Results

The high-risk group had a greater likelihood of noncompliance with antimicrobial therapy completion, as well as subsequent illicit drug use during that time frame, compared with the moderate- and low-risk groups. The low-risk group and most of the moderate-risk group could be safely discharged into the community with PICC lines.

Conclusions

Key in the risk stratification proposal was identifying risk behaviors and determining their degree. Such information provides pivotal delineators in developing risk stratification criteria.

## Introduction

Guidelines on the management of people with intravenous substance use disorders, in clinical settings of severe systemic infectious diseases (IDs), who require parenteral antimicrobial therapy, are limited. Management is a challenge everywhere but especially in under-served rural communities, where resources are scarce, including ambulatory care units, transportation, finance, and even specialists. The available guidelines focus on antimicrobial therapy [1-3], yet offer little or no guidance on the overall treatment of patients who have intravenous substance use disorders. An active use disorder is often the primary cause of the ID and creates an important barrier to the success of the treatment and remission of the ID. Tools are needed to aid providers in identifying who could safely receive outpatient antimicrobial therapy (OPAT) in a clinical setting of intravenous drug use (IVDU) in rural settings.

Approximately 20% of the US population lives in rural areas. Yet, clinical practice in the rural United States can be challenging [4]. Clinical specialists, healthcare facilities, transitional care providers, and substance abuse treatment programs are scarce [5]. Compared with urban dwellers, persons living in rural areas have more health-related disparities. These effects include poorer health, more behaviors that place a person’s health at risk, and limited access to health resources [6]. Substance use disorders, particularly opioid use disorder, have greater prevalence in rural United States [7-10]. In this article, we develop a risk stratification approach similar to approaches used in other disciplines [11-12] to aid rural health care providers in determining which persons with addictive disorders can safely be reviewed for OPAT.

## Materials and methods

 

This study was approved by the Mayo Clinic Institutional Review Board. A retrospective chart review was conducted from January 2014 through July 2016 at Mayo Clinic Health System in Waycross, Georgia, a 200-bed rural hospital with a daily bed occupancy rate of less than 100. It is an affiliate of Mayo Clinic in Jacksonville, Florida. We reviewed all cases that required parenteral, antimicrobial therapy in the outpatient setting as recommended by the ID specialist. The ID specialist screened all patients who may possibly be discharged from the hospital with a peripherally inserted central catheter (PICC) for drug and severe alcohol use. All infected patients who had a positive drug use history and needed a PICC line for discharge were referred for drug addiction consultation. Psychiatric consultations are a routine part of that practice when drug addiction could affect a patient’s outcome. Patients undergo a comprehensive evaluation by a psychiatrist trained in addiction subspecialty and are given the appropriate diagnosis for their addictive disorder, according to the Diagnostic and Statistical Manual of Mental Disorders (Fifth Edition) (DSM-V) [13]. A psychosocial risk profile was created for each case. That profile was used in decision-making around the patient’s disposition, venue of treatment, and recommendations on the ID diagnosis and addictive disorder concurrently. The only inclusion criteria implemented beyond time restrictions were that the person had to have an IVDU disorder, be evaluated by both specialists, and be in need of OPAT. 

Psychosocial risk evaluation

Psychosocial risk evaluations by liaison psychiatrists focusing on patient safety and treatment outcomes are an established practice in assessing the risk levels of candidates for invasive procedures or operations, such as solid organ transplant, left ventricular assist device, pain pump, and deep brain stimulator insertion [11-12]. Such protocols focus on multiple aspects of the psychosocial perspective, including existing psychiatric disease, substance use disorder, lifestyle, treatment adherence etc. In such protocols, each of these aspects is assessed and weighed independently for a specific risk deemed important. For instance, tobacco use disorder is weighed differently in a heart transplant candidate than in a liver transplant candidate whereas alcohol use disorder is of highest importance in a liver transplant candidate. Suicidality or personality features may be of special importance in assessing a candidacy for the insertion of a left ventricular assist device. 

Since no available guidelines for this particular population existed, we reviewed similar, established protocols for psychosocial risk assessment and focused on addictive disorders, psychiatric disorders, psychopathology, and other psychosocial risk factors during the initial, thorough consultation. Obviously, addictive disorders evaluation was the most important aspect of our psychosocial risk formulation when assessing the PICC line compliance. A comprehensive addiction history was obtained focused not only on the primary drug of choice but also other substance use patterns. As expected from standard addiction assessment, the patient was questioned about drug of choice, active use, route of use, longest period of abstinence, previous chemical dependency treatments, relapse prevention skills, and involvement in Alcoholics Anonymous (AA) or Narcotics Anonymous (NA). Insight level was assessed of the person’s addiction and motivation for treatment. After the diagnosis was given, appropriate addiction treatment recommendations were offered in accordance with American Society of Addiction Medicine (ASAM) guidelines [14-15]. Because of the medical acuity in most cases, recommended addiction treatment was either deferred until the end of the antimicrobial course or, in case the decision was made to keep the patient in the hospital, regular follow-up visits by the same psychiatric consultant were completed throughout the hospital stay. During those visits, a dual diagnosis approach was adopted: psychiatric comorbidities were noted and treated where necessary and addiction counseling given. Social support systems, general treatment adherence, living circumstances and financial situation were noted as part of the assessment.

Risk categorization

The retrospective case review was performed. A psychosocial risk profile was formulated for each case and was assigned a risk category of high (HR), moderate (MR), or low (LR) for a peripherally inserted central catheter (PICC) for OPAT.

High risk

Between 72% to 97% of all opioid-addicted persons prefer the oral route for drug administration [16]. Use of an intravenous route is associated with most severe forms of addictive disorders [17-18]. Therefore, all active intravenous drug users within 12 months of the need for OPAT were deemed at high risk for PICC violation without exception. Patients with remote use of intravenous drugs who were in sustained remission but currently deemed at high relapse risk were also assigned to this group.

Twelve months was the length of required abstinence chosen for determining high-risk candidates based on the following considerations.  Most similar existing protocols rely on very limited data on relapse risk prediction, based on a preceding abstinence period [19-22]. We reviewed the literature on all substance use disorders to determine the ideal period of abstinence that would be a reliable indicator for ongoing sobriety. Six months up to two years has been widely considered as the period of required abstinence as relapse predictors in different protocols. Considering our population, we decided on 12 months based on the limited literature and anecdotal data [22]. 

Only 12 months of abstinence from IV substance use disorder without history of formal addiction treatment was adequate to avoid the high risk category but we also carefully looked at individuals who were at high risk of relapse due to a variety of psychosocial factors. To determine high relapse risk in individuals with sustained remission from their addictive disorder, the following factors were considered, most of which are also part of ASAM dimensional assessments [14]: not actively involved in a sober support group (Narcotics Anonymous or Alcoholics Anonymous), active environmental risk factors for relapse (eg, ongoing association with active intravenous drug users), history of recurrent chemical dependency treatment failure, lack of insight or fundamental understanding of addiction, active psychiatric co-morbidity, poor support system and poor financial situation. When unable to confirm the active IVDU or when challenged with poor cooperation, the ID specialist weighed the likelihood that the current clinical infectious situation was caused by active IVDU. When the ID specialist deemed the likelihood high, the case was assigned to the HR group regardless of confirmed use within 12 months. Other considerations factored into the risk profile, including active addiction to other substances (intravenous and non-intravenous patterns), active psychosocial issues (eg, poor support system, poor financial situation), history of poor compliance, and active psychiatric issues. In summary, any patient with a history of IVDU disorder in remission, who had acute concerns for other addictive disorders, psychiatric disorders, or psychosocial issues, was still regarded at high risk for PICC violation.

The HR group was either not approved for discharge from the hospital with a PICC line or were approved to be discharged to a nursing home, a long-term, acute care setting, or an ambulatory infusion center where daily peripheral line insertion is available for all days that therapy is needed. In the rural setting, the decision to keep the HR group in the hospital for the entire course of antimicrobial treatment was made frequently because alternative options were not available. Options such as ambulatory care centers and social services, including transportation, were non-existent in the residential towns of many patients. Patients who involuntarily were unable to adhere to the recommended plan were allowed to be discharged against medical advice. For some severe cases, civil commitment was considered but the local administration was not supportive of this route because the judicial mechanisms to support this option were limited in that particular state.

Moderate risk

Patients were assigned to the MR group if they had a history of intravenous drug use that was in remission for at least 12 months or they had no history of intravenous drug use but had an active opioid, cocaine, or methamphetamine use disorder. The rationale of this assignment was based on the recognition of an untreated addictive disorder, with the possibility of relapse and escalation to an intravenous form of abuse with the availability of the PICC line [23]. Three substance categories received primary focus - cocaine, methamphetamine, and opioids - for this group because these substances are most commonly abused in IVDU [24]. Alcohol or cannabis use disorders were included when they were in severe active form. This rationale was not based on risk of violation of the PICC line but on risk of non-compliance or increased risk of ID relapse during treatment because of active, severe addiction.

The MR group was approved for discharge from the hospital with a PICC line under certain conditions. Patients were allowed to leave with a PICC line but received antimicrobial injections in an ambulatory center at the hospital. For these patients, urine drug screens were randomly conducted. Strict adherence with ambulatory care was required as well as compliance with psychiatry and ID follow-up appointments.

Low risk

Patients without a history of intravenous drug use and in full remission from any other addictive disorder (excluding alcohol or cannabis use disorders in mild or moderate degree) for at least 12 months were assigned to the LR group. This group was cleared for transitioning to OPAT with a PICC line and to routine ID and psychiatric care.

## Results

Between January 2014 and July 2016, 20 cases were identified that were seen by ID services and referred to psychiatry consultation-liaison services for risk assessment and disposition planning (Table 1). Ten cases were assigned to the HR category; five cases, the MR category; and five cases, the LR category. The average age for HR was 38.6 years; for MR, 40.8 years; and for LR, 49.2 years. Of the 20 cases, 12 (60%) were men and eight (40%) were women; in the HR group, seven were men; the MR group, three were women; and in the LR group, three were men. The mean recommended days of antimicrobial therapy for the HR group was 40.2 days (median [range], 42 {10-56} days); for the MR group, 32.2 (median [range], 21 {14-42} days); and for the LR group, 28 (median [range], 28 {14-70}).

**Table 1:** Psychosocial Risk Formulation for OPAT among IV Drug Use Histories Abbreviations: ID, infectious disease; IV, intravenous; OPAT, outpatient antimicrobial therapy; PICC, peripherally inserted central catheter; UDS, urinary drug screen. a. See text for indicators of high relapse risk.

Risk Category	Description	Recommendation
High	Active or suspected IV drug use within 12 months without exception; or drug use in sustained remission >=12 months but currently at high relapse risk.^a^	No approval for discharge home with a PICC line; where possible, discharged to a nursing home, rehabilitation setting, or long-term acute care facility or kept in the hospital for the entire antimicrobial course; if involuntary, discharged home against medical advice.
Moderate	No history of IV drug use but active addiction to methamphetamine, cocaine, opioids, alcohol (only when severe), or cannabis (only when severe).	Approved for discharge from the hospital under restricted conditions: administration of antimicrobial therapy in the hospital ambulatory unit daily with random UDS and strict adherence to recommended psychiatric and ID follow-up appointments.
Low	No history of IV drug use and in full remission from methamphetamine, cocaine, opioids, or active alcohol or cannabis use disorder, or a combination, only of mild or moderate severity.	Cleared for OPAT with a PICC line and appropriate follow-up appointments. No routine UDS required.

In the HR group, an average of 80.3% of IVDU patients completed the antimicrobial therapy days as recommended by ID service; in the MR group, 90.6%; and in the LR group, 100% (Table 2). The most common ID diagnosis for the HR group was native valve endocarditis, followed by epidural abscess, diskitis, facial abscess, and pneumonia. For MR and LR groups, the ID diagnoses were more equally distributed among thigh abscess, septic arthritis, joint arthritis, meningitis, and osteomyelitis.

**Table 2:** Risk Category Demographic Characteristics and ID Diagnosis and Antibiotic Therapy Compliance Abbreviations: F, female; ID, infectious disease; M, male; MSSA, methicillin-sensitive Staphylococcus aureus.

Risk Category	No. of Patients (M/F)	ID Diagnosis, No.	Age, Mean, Median, y	Recommended for Therapy, Mean, Median, d	Compliance With Therapy, Mean (%), Median, d
High	10 (7/3)	Endocarditis, 5	38.6, 42	40.2, 42	32.3 (80.3), 42
		Epidural abscess, 2			
		Diskitis, 1			
		Fascial abscess, 1			
		Pneumonia, 1			
Moderate	5 (2/3)	Thigh abscess, 1	40.8, 34	32.2, 21	29.4 (90.6), 21
		Septic arthritis, 1			
		Pneumonia, 1			
		Prosthetic joint arthritis, 1			
		Osteomyelitis, 1			
Low	5 (3/2)	Endocarditis, 1	49.2, 52	28, 28	28 (100), 28
		Meningitis, 1			
		Soft tissue infection, 2			
		MSSA septicemia, 1			

In the HR group, the most common addictive disorder was intravenous opioid use disorder, which comprised 80% of all HR cases, followed by intravenous cocaine and methamphetamine use disorders (Table 3). Of the 10 patients in the HR group, four (40%) had relapses on the drug of choice during treatment. Among these four patients, two relapsed in the hospital, one in a nursing home, and one following discharge against medical advice. Only one patient in the MR group had a relapse following discontinuation of treatment in an outpatient setting. Violation of the PICC line per se could not be determined. No LR patient had a relapse while receiving OPAT.

**Table 3:** Risk Category and Intravenous Drug Use During Antibiotic Treatment Abbreviation: AMA, against medical advice

Risk Category	Patients, No.	Primary Drug of Choice, No.	Relapse During Treatment	Relapse Venue
High	10	Opioids, 8	4	Hospital, 2
		Methamphetamine, 1		Nursing home, 1
		Cocaine, 1		AMA discharge, 1
Moderate	5	Opioids, 2	1	Home
		Cocaine. 2		
		Cannabis, 1		
Low	5	Opioids, 3	0	
		Cocaine, 1		
		Alcohol, 1		

Four of the 20 patients received more than one diagnosis of an addictive disorder according to DSM-V. These data are detailed in Table 4.

**Table 4:** Risk Category and Diagnostic and Statistical Manual of Mental Disorders (Fifth Edition) Addictive Disorder Diagnoses

	Use Disorder, No. of Patients					
Risk Category	Opioid	Amphetamine	Cocaine	Cannabis	Alcohol	Sedative
High	8	1	3	2	2	3
Moderate	4	0	2	2	0	3
Low	2	1	1	0	3	0
Total	14	2	6	4	5	6

## Discussion

An underlying disorder such as IVDU may lead to ID or an ID may occur unrelated to the addiction. Management of these infections in the IVDU population is a challenge, especially in rural settings with limited resources [5]. One prospective observational study conducted in an urban university hospital in Singapore assessed the care of patients with IVDU who required outpatient intravenous antibiotic therapy [25]. Tamper-proof PICC catheters were used successfully in IVDU patients who self-reported drug use. However, the severity of addiction was not delineated.

Our study is a retrospective review of an ID practice from January 2014 through July 2016 that used an addiction specialist and psychiatrist to aid in the clinical decision- making for the discharge of infected IVDU patients who needed OPAT. Discharge options in the rural setting were scarce. Rarely are board-certified specialists in these two disciplines simultaneously practicing in a small, rural town. The objective of this review was to determine whether a risk stratification criteria aid could be developed to determine who could more safely be discharged home with OPAT. If criteria could be created to differentiate patients who could safely be discharged with OPAT by specialists in a rural community, these criteria could be further tested in a randomized, prospective manner among IVDU persons who were infected and needed OPAT, to aid non-specialist providers in rendering care to this population in small US towns.

We previously reported the cost of hospital care for a recalcitrant IVDU patient who was successfully treated for the infection each time, but because the original problem of intravenous drug addiction was not manageable, multiple new infections resulted [26]. The high non-compliance rate in abstinence from drug use despite being hospitalized in a rural community (the only option available), in the context of the high cost for such care for this population, highlights the gravity of the rural situation and the monumental effect it has on rural hospital finances [27-28]. Our goal was to aid health care providers with a tool to most effectively use limited resources safely. Other disciplines such as organ transplant services allocate resources in an analogous fashion.

We found that the HR group had a greater likelihood of noncompliance with OPAT completion as well as subsequent illicit drug use during that timeframe than the MR and LR groups. Use of illicit drugs during hospitalization occurred despite treatment recommendations for the patient’s addictive disorder and follow-up visits by the psychiatrist during the hospital stay.

All these patients had a life-threatening ID that required parenteral antibiotic treatment. No primary residential addiction program in the area considered them candidates for the addiction programs because of their medical acuity. Use of illicit drugs while being seen by a psychiatrist stresses the degree of the patient’s addiction and the importance of securing the correct psychiatric or addiction diagnosis, or both. This finding highlights that until the primary disorder, addiction, is managed successfully, treatment of secondary problems such as infections is handicapped. Hundred per cent compliance is critical in completing OPAT and not using illicit intravenous drugs during the OPAT timeframe as seen in the LR group. Unfortunately, the numbers in this review are small, a limitation of this study, and yet another reality of reporting from small US towns.

The HR group had more life-threatening infections than the LR group. This fact accounts for the shorter durations of OPAT recommended by ID services. However, it is unlikely to account for the non-existent illicit drug use in the LR group. Opioid use existed in all three risk categories, which emphasizes that the active degree of addiction is the probable predictor of compliance in therapy and safety in OPAT administered via a PICC. The predictor of safety in OPAT discharge likely lies in the adequacy of obtaining an accurate drug use history by a person trained in securing such histories. Many investigators have shown [29-30] that internists and family practitioners often miss the addiction history, let alone accurately determining the degree or severity of the addiction. In this study, two specialists (ID specialist and addiction psychiatrist) simultaneously practiced in a rural community, which is a rare situation. Identification of the risk behavior and its degree are pivotal delineators in developing risk stratification criteria.

## Conclusions

This study provides a risk stratification aid to select those who could safely be discharged with OPAT in US small towns. The severity of the addictions in the HR group and their use of drugs during the treatment period emphasize the enormity of their plight and the lack of options for their care. The proposed HR, MR, and LR risk stratifications of IVDU patients who require OPAT needs further prospective testing with significant numbers to validate the criteria. Such risk stratification of IVDU patients in need of OPAT would afford safer allocation of limited resources, especially in rural locations.
